# Topography and Land Cover of Watersheds Predicts the Distribution of the Environmental Pathogen *Mycobacterium ulcerans* in Aquatic Insects

**DOI:** 10.1371/journal.pntd.0003298

**Published:** 2014-11-06

**Authors:** Kevin Carolan, Andres Garchitorena, Gabriel E. García-Peña, Aaron Morris, Jordi Landier, Arnaud Fontanet, Philippe Le Gall, Gaëtan Texier, Laurent Marsollier, Rodolphe E. Gozlan, Sara Eyangoh, Danny Lo Seen, Jean-Francois Guégan

**Affiliations:** 1 Unité mixte de recherche (UMR) Maladies Infectieuses et Vecteurs: Écologie, Génétique, Evolution, et Contrôle (MIVEGEC) IRD-CNRS-Universities of Montpellier I and II, Centre IRD de Montpellier, Montpellier, France; 2 UMR Territoires, Environnement, Télédétection et Information Spatiale (TETIS) CIRAD, Montpellier, France; 3 Unité d'Epidémiologie de Maladies Emergentes, Institut Pasteur, Paris, France; 4 Ecole des Hautes Etudes en Santé Publique, Rennes, France; 5 Centre de Synthèse et d'Analyse sur la Biodiversité -CESAB. Bâtiment Henri Poincaré, Domaine du Petit Arbois. Aix-en-Provence, France; 6 Bournemouth University, School of Applied Sciences, Dorset, United Kingdom; 7 Service d'épidémiologie et de santé publique, Centre Pasteur du Cameroun, Réseau International des Instituts Pasteur, Yaoundé, Cameroun; 8 Chaire Santé et Développement, Conservatoire National des Arts et Métiers, Paris, France; 9 Institut de Recherche pour le Développement (IRD), UR 072, Laboratoire Evolution, Génomes et Spéciation, UPR 9034, Centre National de la Recherche Scientifique (CNRS), Gif sur Yvette, France et Université Paris-Sud 11, Orsay, France; 10 UMR 912 - SESSTIM - INSERM/IRD/Aix-Marseille Université Faculté de Médecine - 27, Marseille, France; 11 ATOMycA, Inserm Avenir Team, CRCNA, Inserm U892, 6299 CNRS and LUNAM, Angers, France; 12 UMR 207 BOREA IRD-MNHN-Université Pierre et Marie Curie, Muséum National d'Histoire Naturelle, Paris, France; 13 Service de Mycobactériologie, Centre Pasteur du Cameroun, Réseau International des Instituts Pasteur, Yaoundé, Cameroun; University of California San Diego School of Medicine, United States of America

## Abstract

**Background:**

An understanding of the factors driving the distribution of pathogens is useful in preventing disease. Often we achieve this understanding at a local microhabitat scale; however the larger scale processes are often neglected. This can result in misleading inferences about the distribution of the pathogen, inhibiting our ability to manage the disease. One such disease is Buruli ulcer, an emerging neglected tropical disease afflicting many thousands in Africa, caused by the environmental pathogen *Mycobacterium ulcerans*. Herein, we aim to describe the larger scale landscape process describing the distribution of *M. ulcerans*.

**Methodology:**

Following extensive sampling of the community of aquatic macroinvertebrates in Cameroon, we select the 5 dominant insect Orders, and conduct an ecological niche model to describe how the distribution of *M. ulcerans* positive insects changes according to land cover and topography. We then explore the generalizability of the results by testing them against an independent dataset collected in a second endemic region, French Guiana.

**Principal Findings:**

We find that the distribution of the bacterium in Cameroon is accurately described by the land cover and topography of the watershed, that there are notable seasonal differences in distribution, and that the Cameroon model does not predict the distribution of *M. ulcerans* in French Guiana.

**Conclusions/Significance:**

Future studies of *M. ulcerans* would benefit from consideration of local structure of the local stream network in future sampling, and further work is needed on the reasons for notable differences in the distribution of this species from one region to another. This work represents a first step in the identification of large-scale environmental drivers of this species, for the purposes of disease risk mapping.

## Author Summary

Many pathogens persist in the environment, and an understanding of where they are can assist in disease control, allowing us to identify areas of risk to local human populations. Herein, we use general linear models to describe the distribution of a particular environmental pathogen, *Mycobacterium ulcerans*, describing the landscape conditions correlated with the presence of this pathogen in local biota, and mapping the distribution of these habitats in a region of Cameroon, Africa. Our findings identify the importance of the watershed as a factor determining the distribution of the bacterium, where landscape conditions upstream of the sample site can influence the abundance of the bacterium in downstream sites. We find that the bacterium has notable seasonal changes in its distribution, between the wet and dry seasons, which may have implications for human health. We also discuss sensitivity of these models to extrapolation, finding that they work well in the African region and underperforming when extrapolated to another region in South America.

## Introduction

Knowledge of the spatial distribution of an environmentally persistent pathogen is often key in creation of environmental hazard maps for disease control. Yet, despite the importance of this spatial information, only 4% of such pathogens have been mapped [1]. The reason for this gap in our knowledge is practical. It is often difficult to produce large maps of the distribution of these microbial pathogens as they are difficult to detect in nature. A solution to this is to describe the distribution of the pathogens suitable habitat. For example, an environmentally persistent pathogenic bacterium may have a certain *pH* range within which it can survive, a specific range of microaerobic oxygen concentrations [2], and survive preferentially on certain algae [3]. In cases where we have a suitable range of *pH*, a suitable range of oxygen, and suitable algae, we expect to find the bacterium. Herein, this suitable range of microhabitat is termed the ecological niche of the species. Every species in nature, including vectors such as mosquitoes, and pathogens such as *Plasmodium* protozoans, has a unique ecological niche [4], [5].

Knowledge of the distribution of suitable habitats would allow us to predict the expected distribution of the pathogen. This approach has been successfully applied to the vectors of diseases such as malaria, plague and dengue [6], [7], [8], but it is rarely applied to environmentally persistent pathogenic microbes. The range of suitable habitat is, practically, much easier to describe for insect vectors than for microbes. For example, the suitable habitat of mosquitos is driven by factors such as rainfall, which is much easier to describe on a large scale. To describe *pH* in the environment we must visit each site and use a probe at each location. This quickly becomes expensive and time consuming when we consider multiple variables, or if we wish to describe the distribution of a pathogen over large extents.

We hypothesised that these microhabitat variables could be indirectly inferred from large scale macroecological patterns. The distribution of swamp and forested environment, the shape and structure of the landscape, should predict the distribution of these microhabitats. For example, while the suitable habitat of a bacterium may be driven by the suitable combination of *pH*, oxygen, and algae, and other factors, the distribution of these conditions is in turn driven by the landscape. For example, the *pH* and oxygen content of water in swamps is lower, on average, than of water in savannahs. We can use the landscape, which is more easily described, as a proxy to describe the spatial distribution of this suitable microhabitat. Though this approach is limited in lacking a physiological understanding of direct influences on the pathogen, it has the great benefit of inferring the potential distribution of the pathogen, opening new opportunities to disease control.

We undertook ecological niche modelling of *Mycobacterium ulcerans*, an environmentally acquired pathogenic bacterium, and causative agent of Buruli ulcer. The ecological niche refers to this range of conditions within which a species can survive and maintain a population. We infer that, if a species has a large population, it presumably is able to maintain that population, and is in a suitable environment. By understanding the environmental parameters that describe population size, we can predict the distribution of the pathogen. Maps of the distribution of pathogens are often a key step in control of disease, producing environmental hazard maps.

The pathogen of our study, *Mycobacterium ulcerans*, infects up to 10,000 people per year in more than 30 countries around the world [9], [10]. Infection leads to the Buruli ulcer, an emerging neglected tropical disease [10] which results in a necrotizing infection of the skin and can lead to crippling deformity [9]. The transmission route of *M. ulcerans* remains unknown, and though several competing hypotheses exist [11], [12] our work herein does not address transmission, but focuses on the distribution of the pathogen.

Identification of the landscape variants that indicate suitable habitat for this particular pathogen has proven remarkably difficult, despite decades of research (see [13] for a review). Previous research on *M. ulcerans* has found several apparently contradictory facts about the bacterium, making it difficult to establish a generalised picture of its ecology. In 2007 the genome of *M. ulcerans* was sequenced, and analysis revealed extensive evidence for reductive evolution, with massive gene loss. *M. ulcerans* evolved from *M. marinum*, and appears to have undergone a bottleneck event in the process, losing many of the genes *M. marinum* uses to sustain itself in free living environments, apparently now favouring protected environments with low sunlight [14]. This is suggestive of a highly specialised ecological niche, implying that the bacterium cannot survive in a large range of environmental conditions. Detection of the bacterium in the environment is normally via PCR; *M. ulcerans* is very slow growing and extremely difficult to culture from the wild [15], and most attempts at culture result in *M. ulcerans* being overgrown by other bacteria which are ubiquitous in the environment.

However, the implication that the microbe is a specialist has been (apparently) contradicted by recent detection of the bacterium in the environment. *M. ulcerans* DNA has been detected in a bewildering variety of environmental samples, including aquatic insects, biofilms, crustaceans, detritus, fish, frogs, possums and various small mammals, soil, snails, water and worms [3], [15], [16], [17], [18], [19], [20], [21], [22], [23], [24], [25], [26], [27], [28], [29]. This large range of suitable conditions is odd, in light of the bacterium's apparent status as a specialist with a small niche.

The many different species that *M. ulcerans* infects in the local community may become infected due to differences in their feeding habits, position in the trophic web, or relative abundance [13], [30], [31]. Herein, we use samples of the five dominant Orders of the aquatic insect community, which have been tested for *M. ulcerans* positivity rates, and correlate changes in *M. ulcerans* positivity in these 5 Orders to changes in the environmental conditions of land cover and topography. These 5 Orders may not be the primary habitat of *M. ulcerans* in the wild, as the full biotic extent of *M. ulcerans* distribution is still unknown, but they are commonly found to be persistently infected and appear to be important hosts [32]. Previous work has found that *M. ulcerans* abundance does respond to water body type, being more commonly detected in swamps (still lentic systems) than rivers (flowing lotic systems) in Ghana [33], [34]. The pathogen is associated with lowland, flat, swampy areas in contact with stagnant water [35], is known to have complex seasonal dynamics [32], and appears to be present at low levels throughout the entire local biotic community along the year [29]. The distribution of the disease may also inform us on the distribution of the pathogen; the distribution of Buruli ulcer is known to be more spatially restricted than the distribution of *M. ulcerans* [36], and is known to respond to low elevation, forested land cover, and previous rainfall [37], [38], which would suggest that perhaps these factors are also important in the distribution of *M. ulcerans*. Taken together, these facts suggested that changes in the biotic distribution of the pathogen could be mapped using landscape variables. Often, sampling of river systems results in the unexpected presence of *M. ulcerans*; if factors at the larger watershed scale add substantial information on the distribution of *M. ulcerans* a description of the upstream region of the river may help to explain this unexpected presence. We describe the condition of the landscape using land cover, such as forest and savannah, and topography, such as elevation and slope. These landscape scale factors are expected to indirectly influence *M. ulcerans* abundance via their influence on the microhabitat the bacterium inhabits, for example affecting the *pH*, dissolved oxygen content, and composition of the aquatic insect community, which are known to influence *M. ulcerans* distribution [12], [29].

To address our questions we describe landscape variables correlated to the presence of the bacterium in aquatic macroinvertebrates in Cameroon, Central Africa. We then test our model against data collected in French Guiana to explore the generalizability of our findings. This will contribute to an understanding of the spatial distribution of this environmental pathogen, and further our ability to control Buruli ulcer disease.

## Materials and Methods

A model was constructed on the dataset from Akonolinga, Cameroon, and predicted into French Guiana, South America. This enabled us to describe the niche of *M. ulcerans*, and examine how well these models transferred to other areas.

### Study sites, sampling methodology and response variable

**Figure 1:**
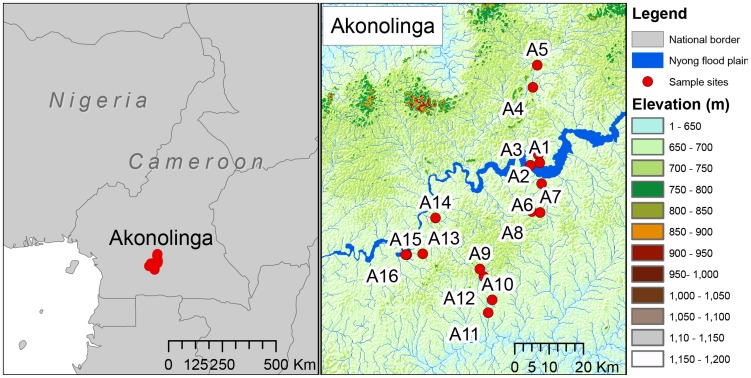
Location of sample sites in Cameroon, as in [30]. Within Cameroon, Akonolinga is almost entirely rainforest. This region is dominated by the Nyong river and has fewer highland areas. Red dots are sample sites in Akonolinga.

The Cameroon dataset is a subset of that published in [29], which comprises 16 sites in Akonolinga, sampled every month for 12 months (Figure 1). Identical methods were carried out by the same investigators for all sites throughout the study. In brief, at each site, 4 locations were chosen in areas of slow water flow and among the dominant aquatic vegetation and at each location, 5 sweeps with a dip net within a surface of 1 m^2^ were done to sample the aquatic community. Aquatic organisms were classified down to the Family level whenever possible and stored separately in 70% ethanol. Individuals belonging to the same taxonomic group were pooled together for detection of *M. ulcerans* DNA by quantitative PCR. Among these, the 5 most abundant Orders (Diptera, Hemiptera, Coleoptera, Odonata and Ephemeroptera) were consistently analysed for all sites and months. Pooled individuals were all ground together and homogenized and DNA from tissue homogenates was purified using QIAquick 96 PCR Purification Kit (QIAGEN). Finally, amplification and detection of MU DNA were performed through quantitative PCR by targeting the ketoreductase B domain (KR) of the mycolactone polyketide synthase and IS2404 sequence from MU genome. This resulted in 5 analyzed samples (each Order) per month, per site, which we use to infer *M.ulcerans* presence or absence. Summary statistics are described in Table 1. Sampling effort varied from month to month, as is discussed in [29], however we have used a subset of that data in order to gain the most consistent representation of the biotic community possible.

**Table 1:** *M. ulcerans* distribution at sample sites in Akonolinga, Cameroon.

				Wet season		Dry season	
Site Code	Latitude	Longitude	Type of water body	Relative abundance of the 5 Orders (%)	PCR positive samples of the 5 Orders,% (positive/samples)	Relative abundance of the 5 Orders (%)	PCR positive samples of the 5 Orders,% (positive/samples)
A1	N3 46.806	E12 16.133	Swamp	93.69	6.98% (3/43)	97.39	20% (4/20)
A2	N3 47.083	E12 15.383	Swamp	94.69	12.36% (11/89)	94.53	8.11% (3/37)
A3	N3 46.316	E12 14.440	Stream	90.82	2.56% (1/39)	90.41	0% (0/20)
A4	N3 58.464	E12 14.796	River	55.12	5.13% (2/39)	32.17	5.26% (1/19)
A5	N4 02.255	E12 15.620	Stream	83.64	10% (4/40)	75.30	5.26% (1/19)
A6	N3 43.483	E12 16.466	Swamp	90.31	17.24% (15/87)	85.85	2.94% (1/34)
A7	N3 38.889	E12 15.986	River	79.52	10.26% (4/39)	79.13	0% (0/13)
A8	N3 38.980	E12 14.696	River	64.86	9.09% (4/44)	73.89	7.69% (1/13)
A9	N3 29.912	E12 06.425	Swamp	82.40	4.4% (4/91)	91.67	2.7% (1/37)
A10	N3 29.912	E12 06.425	Swamp	93.62	7.5%(3/40)	89.90	0% (0/20)
A11	N3 23.271	E12 07.870	River	50.12	4.65% (4/86)	59.56	8.82% (3/34)
A12	N3 28.788	E12 07.255	River	52.87	2.44% (1/41)	66.19	15% (3/20)
A13	N3 32.322	E11 57.643	Swamp	81.92	2.22% (2/90)	79.39	8.11% (3/37)
A14	N3 38.032	E11 59.695	River	64.77	15.38% (6/39)	63.37	20% (4/20)
A15	N3 32.288	E11 55.239	Flooded	94.33	11.11% (4/36)	97.54	0% (0/15)
A16	N3 32.276	E11 55.181	Stream	89.85	2.86% (1/35)	93.40	0% (0/15)

16 sites were sampled for 12 months, sampling from different types of water bodies. The dominant members of the aquatic biota were Diptera, Hemiptera, Coleoptera, Odonata and Ephemeroptera. These made up the majority of the community in both seasons; the percentage of the biotic sampled community composed of these five groups is reported as Relative abundance of the 5 Orders in Table 1. These communities were normally positive of *M. ulcerans*, the percentage of positive samples (number of positive samples/total samples for the 5 Orders from that site in that season) describes the PCR positive samples of the 5 Orders. This table is a summary of a subset of the data presented in [30].

A data set following the same methodology was independently collected in French Guiana, South America [28]. DNA extraction was carried out with the same two primer pairs and methodology as above. In French Guiana eighteen sites were sampled twice during the wet season, which lasts from December to July. The entire biotic community was sampled, and for consistency the same 5 taxonomic Orders as in Akonolinga (Table 2) were compared.

**Table 2:** *M. ulcerans* distribution at sample sites in French Guiana, South America.

				Wet season
Site Code	Latitude	Longitude	Relative abundance of 5 Orders (%)	PCR positive samples of the 5 Orders,% (positive/samples)
FG10	N4 44.170	W-52 19.618	58.62	44.12% (15/34)
FG11	N4 50.284	W-52 21.195	52.54	32.26% (10/31)
FG19	N5 17.773	W-53 03.085	62.50	10.00% (1/10)
FG2	N5 37.888	W-53 42.433	70.83	11.76% (6/51)
FG23	N5 21.724	W-53 2.0200	29.27	16.67% (2/12)
FG28	N5 36.328	W-53 49.660	56.60	20.00% (6/30)
FG34	N4 50.068	W-52 18.126	85.00	32.35% (11/34)
FG38	N5 23.646	W-52 59.521	73.74	13.70% (10/73)
FG41	N5 25.725	W-53 05.326	41.07	8.70% (2/23)
FG43	N5 22.632	W-52 57.232	75.47	2.50% (1/40)
FG44	N4 20.052	W-52 09.148	25.42	0.00% (0/15)
FG45	N4 18.025	W-52 07.397	61.95	2.86% (2/70)
FG46	N5 02.121	W-52 30.989	74.24	2.04% (1/49)
FG47	N4 55.744	W-52 24.229	65.00	0.00% (0/26)
FG48	N4 51.616	W-52 16.518	16.67	0.00% (0/1)
FG49	N5 39.996	W-53 46.794	36.54	0.00% (0/19)
FG53	N5 36.136	W-53 50.182	67.86	0.00% (0/57)
FG7	N4 51.648	W-52 15.405	29.41	0.00% (0/10)

18 sites were sampled in the wet season. The dominant members of the aquatic biota were Diptera, Hemiptera, Coleoptera, Odonata and Ephemeroptera, as in Akonolinga. These made up the majority of the community, the percentage of the biotic sampled community composed of these five groups is reported as Relative abundance of the 5 Orders. These communities were normally positive of *M. ulcerans*, the percentage of positive samples (number of positive samples/total samples for the 5 Orders from that site in that season) describes the PCR positive samples of the 5 Orders. This table is a summary of a subset of the data presented in [28].

### Seasonal effects on *M. ulcerans* distribution

*M. ulcerans* has previously been found to respond to variables that are influenced by rainfall [35], [38]. To explore differences in the seasonal distribution of the bacterium, the wet season months and the dry season months were analysed separately. In Cameroon wet season months are April, May, June, August, September and October. The dry season is January, February, March, July, November and December. For each site, the proportion of positive samples at a site in a season was determined by summing the number of positive samples in that season, then dividing by the total number of samples sampled in that season (which is 5 multiplied by the number of sampled months). This resulted in two response variables, Y_wet_ and Y_dry_, which we use to describe the proportion of *M. ulcerans* positive samples in the 5 dominant insect Orders in the wet and dry seasons respectively. This resulted in a general, standardised view of the mycobacterium distribution in both the dry and wet seasons. The habitat suitability is determined by the proportion of samples of the biotic community that are *M. ulcerans* positive.

### Land cover and topography

Land cover in Akonolinga was described using several multispectral satellite images; SPOT 2.5 meter resolution images (references: 50833380811220923092V0 and 50833371012210937422V0), and a Landsat image (reference L72186056_05620021107). The study area was categorised into the following classes; Agriculture, Forest, Flood plain, Road, Savannah, Swamp and Urban (Table S1). Classification was conducted in the Object Orientated Image Analysis software eCognition [39]. The resulting maps were validated and corrected where needed following onsite visits in November 2012. Topography was described using the Shuttle Radar Topography Mission (SRTM) digital elevation model [40], which has a spatial resolution of 90 meters. All topographical variables were derived using the Spatial Analyst extension of the software ArcMap 10.1 [41]. For each site we described the mean, standard deviation, minimum, maximum and variety of elevation, in meters above sea level, using SRTM (Table S1). From the SRTM we calculated the mean, standard deviation, minimum, maximum and variety of the topological slope, in degrees. Flow accumulation is the accumulated number of upstream cells flowing into a point, and ecologically represents the topographical potential for water to accumulate. We derived the mean, standard deviation, maximum and variety of the flow accumulation. We also calculated mean, standard deviation, maximum depth, variety, and proportion of buffer surface area covered by basins. Basins are depressions in the landscape where water is expected to accumulate and, potentially, stagnate, and were detected using the Fill function in Spatial Analyst extension in Arc Map. Stream order indicates the distance from the source of the river, and is a simple index of the type of stream (1^st^ order being small streams, larger orders being big rivers). Proportion of 1^st^ to 8^th^ order streams, defined by Strahler method [42], was recorded in each buffer. Finally, wetness index is the topographic potential for water to accumulate. It was derived from the flow accumulation and the slope, according to the Equation 1, where WI is the wetness index [43], FA is flow accumulation and S is the topographic slope in degrees. We derived the mean, standard deviation, maximum, and variety of wetness index values, and the proportion of buffer surface area covered by wetness index values which are positive (relatively wet areas) and negative (relatively dry areas).
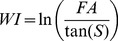
(1)


### Importance of local effects compared to regional effects in *M. ulcerans* distribution

**Figure 2:**
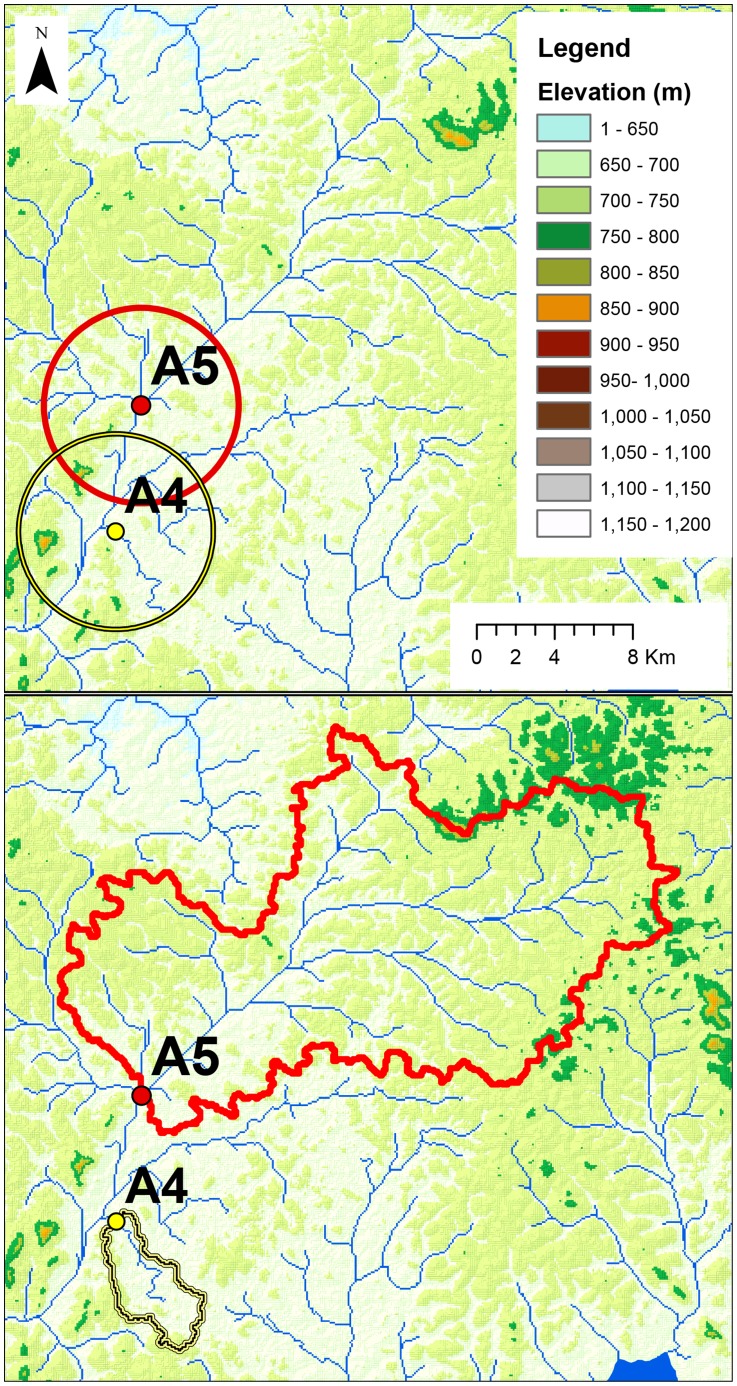
An example of the two buffer types used in this examination, sites A4 and A5 in Akonolinga. This is in the north of Akonolinga, near the village of Emvong. The upper panel is a 5 km buffer around the sites, within this region we describe the topography and land cover, and its association with *M. ulcerans* abundance. We compare this to the watershed buffer (lower panel). The watershed is the drainage area for each site, in principle all water that falls within this region will eventually pass through the sample site.

The topography and land cover of the sample sites were described within two different buffers (Figure 2). These buffers corresponded to local and regional conditions. The first buffer was a 5 km radius circle around the sample site, which was chosen to represent the local conditions. 5 km is, approximately, the flight range of the 5 insect orders sampled [44], [45], [46], [47]. The insects should be able to move throughout this region, be exposed to *M. ulcerans*, before being captured at the sample site. We describe the land cover and topography within this 5 km buffer and correlate the condition of this region to the proportion of *M. ulcerans* positive pools in each season.

The second buffer was defined using the watershed of the sample site (Figure 2). The watershed is the upstream catchment area. In principle, all water within this region, and any detritus floating in the water, will eventually flow through the sample site. Watersheds can vary greatly in size, easily being several kilometres long, and detritus from very distant locations can flow quite large distances. *M. ulcerans* is known to attach to such detritus [24]. This watershed buffer is created using the Watershed tool in ArcMap10.1, Spatial Analyst extension [42].

### Principal component analysis

The 42 variables estimated to describe the landscape were reduced to permit modelling. Principal component analysis (PCA) was performed on the landscape variables centred at the mean (*ln*(x)−*ln*(x_mean_)) to summarize the data in the watershed and the 5 km buffer. PCAs were performed with the PCA function in the FactoMineR library in R [48]. This generated two PCAs; a PCA of the 42 environmental variables in the watershed buffer, PCA_ws_, and a PCA of the 42 environmental variables in the 5 km buffer, PCA_5 km_. In each PCA we examined the orthogonal axes that explained 95% of the variance in the 42 topography and land cover variables.

Firstly, 9 principal components explained 95% of the variance in the watershed of the sample site (PCA_ws_). The magnitude and direction of each correlation is given in the supplementary materials (Tables S1 and S2). We describe PCA_ws_1 as “large watersheds that drain flood plains”, given its strongly positive correlations to watershed surface area and floodplains; PCA_ws_2 as “large watersheds that drain highland agriculture”; PCA_ws_3 as “large watersheds that drain lowland agriculture”; PCA_ws_4 as “small watersheds that drain swamp and forest at flat intermediate elevations”; PCA_ws_5 as “small watersheds that drain highland urban and savannah”; PCA_ws_6 as “small watersheds that drain highland urban and forest”; PCA_ws_7 as “large watersheds that drain lowland forest, savannah and swamp”; PCA_ws_8 as “small watersheds that drain urban and agricultural environments in hilly lowlands”; and PCA_ws_9 as “small watersheds that drain wet swamps in areas that reach from low to high elevations” (Table S1).

Secondly, for the local 5 km circular buffer, 6 principal components (PCA_5 km_) explained 95% of the variance in the data as described in SM2. Translating these to ecologically meaningful terms, we describe PCA_5 km_1 as representing “sites surrounded by flat lowland areas with urban, agriculture and the flood plains of large rivers”; PCA_5 km_2 as representing “sites surrounded by sloped highland areas with urban, agriculture and small rivers”; PCA_5 km_3 as representing “sites surrounded by sloped highland areas with savannah and large swampy rivers”; PCA_5 km_4 as representing “sites surrounded by flat lowland areas with savannah and small rivers”; PCA_5 km_5 as representing “sites surrounded by flat highlands with urban, agriculture and large rivers”, and PCA_5 km_6 as representing “sites surrounded by lowland hills, with small rivers and many small basins, in unforested environment”, (Table S2).

### Model fitting and evaluation

We allow model selection to choose which of these principal components are most informative in the species distribution, Y_wet_ and Y_dry_. The dry season general linear models (GLMs) and wet season GLMs were fitted separately with glmulti in the glmulti library in R. Glmulti finds the best set of GLMs among all possible combinations of explanatory variables; so for example all possible Y_dry_∼PCA_5 km_ models were fitted, and each was evaluated with the Akaike information criterion corrected for small sample sizes (AICc). Low AICc scores indicate good performance and reduced overfitting [49]. The best set of these binomial GLMs (within 2 AICc scores of the best model) are selected, and the model within this range with the lowest sum of absolute residuals (best performance) is selected as the final model (Figure S1).

The response variable changed seasonally, resulting in two response variables, Y_dry_ and Y_wet_. Along with the PCA_5 km_ and PCA_ws_ inputs this resulted in four models; Y_dry_∼PCA_5 km_ and Y_dry_∼PCA_ws_ in the dry season, and Y_wet_∼PCA_5 km_ and Y_wet_∼PCA_ws_ in the wet season. This reduces our variables by retaining those that are important. Then, to compare the importance of PCA_5 km_ (local) and PCA_ws_ (regional watershed) in the distribution of the response variable, *M. ulcerans* abundance, the components retained in these models were included in the final models, Y_dry_∼PCA_5 km_+PCA_ws_ in the dry season, and Y_wet_∼PCA_5 km_+PCA_ws_ in the wet season. In this way, by allowing glmulti to retain or drop these variables we can compare the importance of the watershed and local 5 km area variables in the distribution of *M. ulcerans*.

Potential effects of multicolinearity were explored but were deemed minimal, as all pairwise Pearson correlation coefficient R values in the principal components were below 0.75 (Tables S3 and S4).

In the initial screen of variables, Y_dry_∼PCA_5 km_ and Y_dry_∼PCA_ws_ retained PCA_ws_4, “small watersheds that drain swamp and forest at flat intermediate elevations”, PCA_ws_9, “small watersheds that drain wet swamps in areas that reach from low to high elevations” and PCA_5 km_2, “sites surrounded by sloped highland areas with urban, agriculture and small rivers”. These were included in the model of interest, Y_dry_∼PCA_5 km_+PCA_ws_.

For the wet season Y_wet_∼PCA_5 km_ and Y_wet_∼PCA_ws_ retained PCA_ws_1, “large watersheds that drain flood plains”, PCA_ws_ 5, “small watersheds that drain highland urban and savannah”, PCA_ws_ 6, “small watersheds that drain highland urban and forest”, PCA_ws_ 8, “small watersheds that drain urban and agricultural environments in hilly lowlands”, PCA_5 km_2, “sites surrounded by sloped highland areas with urban, agriculture and small rivers” and PCA_5 km_4, “sites surrounded by flat lowland areas with savannah and small rivers”, which were included in Y_wet_∼PCA_5 km_+PCA_ws_.

### Predicting the spatial distribution of suitable habitat for *M. ulcerans* in the model training region, Akonolinga

We interpolate the Akonolinga model within the region of Akonolinga to predict the distribution of suitable habitat, the reservoir, of *M. ulcerans*. To achieve this, points where streams (defined using STRM) flow under or across roads (defined using satellite images) were selected. These were termed ‘pour points’ in this article. Selection of the point where streams cross roads was based on the hypothesis that these environments, where contact between humans and the aquatic environment will be high, may be important in infection. This does not mean that infection does not occur in other locations, nor do we speculate on the importance of relative routes of transmission. This will not characterise all the environmental reservoir of the bacterium, but will describe an important part of it. The topography and land cover of the watershed and 5 km buffer of these pour points was characterised, transformed into PCA_5 km_ and PCA_ws_ format, and the GLM was predicted. As a summary to describe this distribution, we use Morans Index of spatial autocorrelation, which describes the extent to which the distribution is random, and is here used to describe the distribution of suitable sites. This is implemented using the tool Spatial Autocorrelation Global Moran's I in ArcMap10.1 [41].

### Predicting the spatial distribution of suitable habitat for *M. ulcerans* in a new region, French Guiana

We extrapolate the Akonolinga wet season model to French Guiana, to understand how the suitable habitat in one region is similar to that in another. For comparability, the wet season model, constructed in Cameroon, was used to predict the positive sites among the 18 sampled sites in French Guiana. Values of PCA_5 km_ and PCA_ws_ in French Guiana were generated using the ind.sup option in the PCA function. The Akonolinga wet season model was then predicted into French Guiana using the land cover data provided by the French *Ministère de l'Écologie, du Développement Durable et de l'Énergie* [50], and topography derived from SRTM.

As discussed above, the choice of error structure is important in the performance of a GLM. We aim to describe the distribution of the bacterium, so preference is given to the model with the lowest residual values in the model, which in this case is Gaussian rather than Binomial error structure. Residuals were much lower in a Gaussian model, as shown in Figures S2 and S3 (see the observed response versus predicted response for Gaussian and Binomial models and QQ plots for the Gaussian and Binomial models, respectively). This difference is an order of magnitude. This was a practical decision – using Gaussian models in this case was based entirely on the desire to clearly predict where this pathogenic bacterium is more likely to occur, in such a case errors of residuals have a greater cost.

The wet and dry season watershed Gaussian models were predicted on the pour point data using the predict.glm function in R. The model predictions of habitat suitability at these pour points were then interpolated using Inverse Distance Weighting in the IDW tool of ArcMap 10 [41].

## Results

### Relative importance of local and regional effects on the distribution of *M. ulcerans* in wet season

The final fitted wet season Binomial logit GLM, after stepwise AICc selection, was
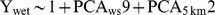
The final GLM suggested that both local and regional effects are substantially correlated to *M. ulcerans* distribution. Regional effects were represented by PCA_ws_9, “small watersheds that drain wet swamps in areas that reach from low to high elevations”, and was negatively correlated to *M. ulcerans* abundance (correlation coefficient −0.37, *p* = 0.007). This means we expect less *M. ulcerans* in small watersheds that drain swamps near highlands. The second part of the above equation corresponds to local effects; PCA_5 km_2 represents “sites surrounded by sloped highland areas with urban, agriculture and small rivers”. This was also negatively correlated to *M. ulcerans* abundance (correlation coefficient −0.16, *p* = 0.00214), so we expect less *M. ulcerans* when the area around the sample site is highland areas with urban and agricultural areas.

**Figure 3:**
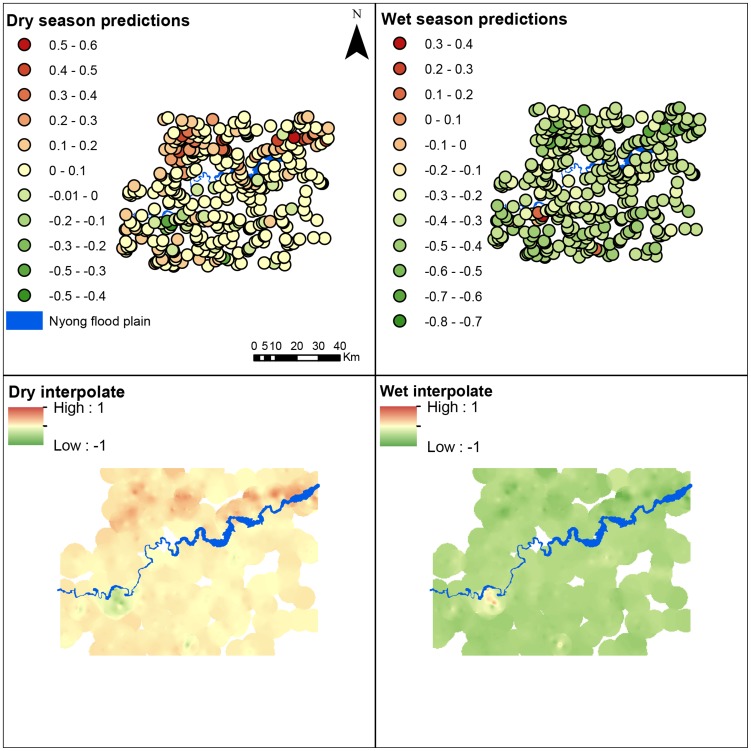
Spatial distribution of habitat suitable for *M. ulcerans* in Akonolinga, Cameroon. Units of habitat suitability are the proportion of qPCR pools predicted to be positive, based on the field work of [30]. Negative values are a result of the normal distribution of the residuals (Figures S4 and S5). The Gaussian wet and dry season models, based on the original 16 sites, are predicted into each of the pour points (where a stream crosses a road) in the region (top row), resulting in the predicted habitat suitability at each point. The pour points are interpolated (bottom row) using IDW fixed distance 0.05 decimal degrees interpolation (ArcMap10.1) resulting in the first map of spatial distribution of *M. ulcerans* encounter risk.

The spatial distribution of *M. ulcerans* suitable habitat in the wet season predicted at the pour points was non-random, based on Moran's I spatial autocorrelation (Moran's Index: 0.21, z-score: 9.1, *p*<0.00001), positive sites tend to cluster together (Figure 3).

### Relative importance of local and regional effects on the distribution of *M. ulcerans* in dry season

The final fitted dry season binomial logit GLM, after stepwise AICc selection, is
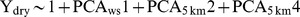
The final models on the dry season found that both regional and local effects were substantially correlated to presence of *M. ulcerans*. Regional effects were represented by PCA_ws_1, “large watersheds that drain flood plains”, which was marginally negatively correlated to *M. ulcerans* abundance (correlation coefficient −0.26, *p* = 0.05210). PCA_5 km_2, “sites surrounded by areas with urban, agriculture and small rivers” was positively correlated to *M. ulcerans* abundance (correlation coefficient 0.09, *p* = 0.18709) though the *p* value suggests this is not significant, and finally PCA_5 km_4, “sites surrounded by areas with savannah and small rivers”, was positively correlated to *M. ulcerans* abundance, (correlation coefficient 0.38, *p* = 0.007).

The spatial distribution of *M. ulcerans* suitable habitat in the dry season predicted at the pour points is non-random, based on Moran's I spatial autocorrelation (Moran's Index: 0.33, z-score: 14.32, *p*<0.00001) positive sites tend to cluster together (Figure 3).

### Model performance when interpolated in Akonolinga

Spatial autocorrelation of model residuals can be an issue in GLMs, but this was explored, and it was not the case here. Model residuals were not significantly spatially autocorrelated in the wet season (Moran's Index: −0.285386, z-score: −1.045844, *p* = 0.295633) nor in the dry season (Moran's Index: 0.071225, z-score: 0.655435, *p* = 0.512187).

The AICc of the final dry season Binomial model was 49.6, the absolute sum of the residuals was 11.03. The AICc of the final wet season Binomial model was 67.8, the absolute sum of the residuals was 11.95.

We note that Gaussian models had significantly better performance. The AICc of the final dry season Gaussian model was −39.8, the absolute sum of the residuals was 0.53. The AICc of the final wet season Gaussian model was −65.5, the absolute sum of the residuals was 0.24. Model performance is presented in Figure S2, model residuals were normally distributed (Figure S3).

### Model performance when extrapolated in French Guiana

**Figure 4:**
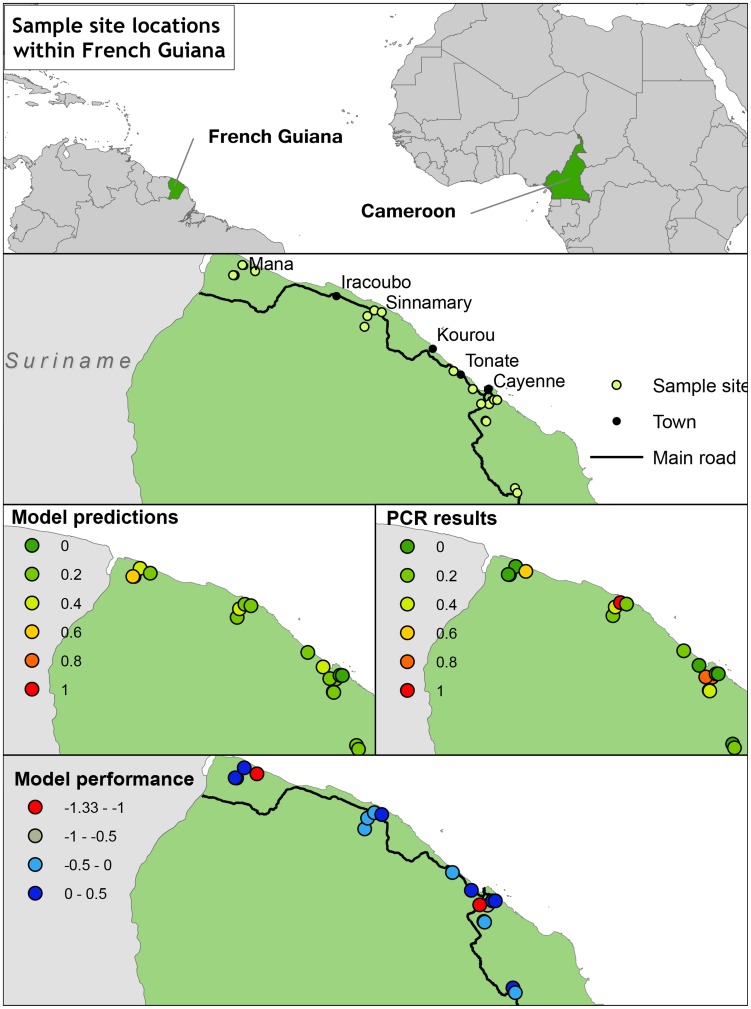
Model validation in French Guiana. Sample sites were as in [28]. A wet season Gaussian niche model based on data collected in Cameroon was predicted into French Guiana (3^rd^ row, left hand side). The model under-predicted, *M. ulcerans* was present in more sites than expected (bottom row, model residuals). A similar Binomial model predicted all sites to be negative.

The Akonolinga wet season model was predicted into 18 sample sites in French Guiana (Figure 4, 2^nd^ row). The model predicted sites to be positive or negative, and the results of qPCR corroborated these predictions (Figure 4). Performance of the Binomial model was notably poor, all sites were predicted negative. In contrast, performance of the Gaussian model was better, but accuracy was still poor at 0.39 (Table S5). Sensitivity and negative predictive values are high, indicating that the predictions of presence of the bacterium are likely to be true, specificity and positive predictive values are low; indicating predictions of absence of the bacterium are likely to be incorrect. This is a result of a bias towards Type II errors (false negatives) in the Gaussian model. Overall, the model predicts *M. ulcerans* in Akonolinga, but is sensitive to extrapolation. Extrapolation tends to result in false negative predictions of presence.

## Discussion

Here, we have demonstrated that in addition to local variables around the sample site, the distribution of *M. ulcerans* correlates to regional variables, i.e. the topography and land cover of the watershed of the sample site. This spatial distribution of suitable habitat was described, allowing the production of environmental hazard maps for the distribution of the pathogen. *M. ulcerans* presence in the wet season correlates with lowland areas surrounded by few agricultural or urban areas, particularly if the sample site has a large watershed. We expect more *M. ulcerans* in the dry season in sites surrounded by urban and agricultural areas, with many small streams, particularly if the sample site has a small watershed.

Many of the findings are in accord with what little we already understand about this bacterium. *M. ulcerans* has been previously associated with flat wetland areas [9], [35]. A similar association with Buruli ulcer has been reported [51], which found that high standard deviation of the wetness index was a risk factor for Buruli ulcer. These three variables are normally strongly correlated to each other and ecologically similar entities. In this study these are negatively correlated to PCA_ws_9, here termed “small watersheds that drain wet swamps in areas that reach from low to high elevations” which negatively correlated to *M. ulcerans* abundance: these studies appear to be describing the same ecological entity, but with different variables.

Our study was limited in certain regards, as we focused it on the prevalence of *M. ulcerans* in the biotic community, and on how topography and land cover in the region could influence that prevalence. We do not consider abiotic conditions testing positive for *M. ulcerans*. Potentially the abiotic distribution may respond differently to these variables, future work will aim to explore this. However, given that *M. ulcerans* is commonly detected in the biotic environment and appears to be at lower prevalence in the abiotic environment, we believe our results are still applicable to an understanding of *M. ulcerans* distribution. We had a relatively low positivity rate (Table 1). A potential limitation is that low positivity can bias a model towards false negatives, while this is possible we are unable to test this further with our current data.

The Akonolinga wet season model was extrapolated into French Guiana, where sampling was in the wet season. Despite good performance in Akonolinga, the model performed poorly in French Guiana, under-predicting the bacterium's distribution (Figure 4). There are a number of points to be drawn from this. First, there were differences in sampling effort between the two sites, as the Akonolinga sampling regime consisted of 12 time points in the year, while the French Guiana regime consisted of 2 time points. This would be consistent with the idea that the bacterium is transiently present in different regions, and under-prediction would be expected in this case. Secondly, a potential complication results from differences in the ability of the SRTM dataset to delineate watersheds due to dense rainforest canopies in French Guiana [52]. The shape of a watershed is sensitive to the quality of the elevation data used, errors in the digital elevation model, or man-made drainage structures, can have effects not captured by this model. Finally, we cannot rule out that the differences are a result of differences in *M. ulcerans*. We used qPCR to detect *M. ulcerans*, however the species is known to have multiple ecovars [53], [54] and subspecies, distributed differently throughout the globe. If it is the case that we are predicting the ecological niche of one Akonolinga *M. ulcerans* species into French Guiana, and testing it against a separate French Guiana species, one would expect the model to under-predict if the French Guiana subspecies occupies a larger ecological niche.

Regardless of error structure, selection of both types of models (Gaussian and Binomial) retained watersheds as important variables. These findings will impact future research on Buruli ulcer and *M. ulcerans*; future sampling regimes would benefit by consideration of the local hydrology before beginning sampling, and selecting sample sites along these lines. We also postulate the importance of watersheds as a barrier to dispersal for the bacterium. A recent key study found a strong relationship between *M. ulcerans* population structure and the greater West African hydrological watersheds [53], with populations being bound to watersheds. These are the drainage areas of large rivers such as the Nyong, Mbam and Ouémé rivers, a much larger scale than our study. However, given our results herein, it seems the bacteria may drift downstream. This is inferred by the difference in the effect of watershed size from dry to wet seasons.

This is consistent with the idea of a ‘flushing’ effect of rainfall in the wet season, carrying bacteria downstream [38], which will influence their genetic population structure. This has notable consequences for the epidemiology of Buruli ulcer. If the watersheds are barriers to movement for the bacteria it implies that *M. ulcerans* may be common in the environment, but in certain areas hydrological conditions facilitate concentration of the bacterium, as is the case with anthrax [55].

### Conclusion

The distribution of environmental pathogens needs to be understood to facilitate control. Commonly, local effects in the microhabitats are considered to describe the ecological niche of a pathogen. However our study demonstrates that regional effects are important factors to be considered. Future research on the *M. ulcerans* would benefit by considering the watershed of potential sample sites, particularly as such data is often quite simple to acquire. The shape, size, and land cover of the watershed correlates with changes in the distribution of *M. ulcerans*, and useful information is lost if watersheds are ignored. The distribution of swamp in a watershed was found to be an important factor in the suitability of the site for *M. ulcerans*; though a sample point in the field may be at a location normally considered unsuitable for the bacteria (e.g. a small swift lentic stream), the area upstream may contain an abundance of lotic swamps and be quite suitable for the bacterium, which may be ‘washed out’ downstream towards the sample site. This is an example of the useful information we gain by placing pathogens in an environmental context, rather than regarding them solely in an epidemiological sense.

## Supporting Information

Figure S1 GLMulti output, for binomial and Gaussian models. Sum of absolute model residuals are plotted against AICc. Within the region of 2 AICc scores of the best model (vertical lines) we select the model with the lowest residuals (highlighted in red).
(DOC)

Figure S2 Observed against predicted values for each model. Note that Gaussian models have a much better fit.
(DOC)

Figure S3 Quantile-quantile plots of normality. The Gaussian and Binomial are both similarly normally distributed, though the Binomial displays a larger variance of residuals.
(DOC)

Table S1 Results of principle component analysis for topographical and land cover variables in a watershed buffer. 95% of the variance in the data was described with 9 components, the eigenvalue of each component is given at the bottom of the table. Each component correlates differently to different variables, red highlights negative correlations, blue highlights positive correlations. PCA_ws_1 describes large watersheds that drain flood plains and swamps, with few urban and agricultural areas. These are high elevation areas with variable slopes. PCAws2 describes large watersheds that drain agriculture at flat highland areas. PCA_ws_3 describes large rivers that drain urban and agriculture areas at flat lowlands with, with little forest. PCA_ws_4 describes small rivers, with small watersheds that drain forest and swamp areas, without urban areas. These are at intermediate elevations, with flat areas. PCA_ws_5 describes small rivers that drain urban and savannah areas, predominantly in higher elevation flat lands. PCA_ws_6 corresponds to small low order streams that drain urban and forest (not agriculture) in high elevation slopes. PCA_ws_7 is larger watersheds that drain forest, savannah flood plain and swamp, in areas with flat, wet, lowlands. PCA_ws_8 represents small watersheds that drain urban & agriculture, flood plain and savannah. These areas are wet lowlands with lots of small hills. PCA_ws_9 represents small watersheds that drain wet swamps in areas that reach from low to high elevations.
(DOC)

Table S2 Results of principle component analysis for topographical and land cover variables in a 5 km buffer around the sample site. 95% of the variance in the data was described with 6 components. Each component correlates differently to different variables, red highlights negative highlights, blue indicates positive correlations. Surface area is constant, at π5^2^ = 79 km^2^. PCA_5 km_1 represents sites surrounded by flat lowland areas and urban, agriculture and the flood plains of large rivers. PCA_5 km_2 represents sites surrounded by sloped highland areas and urban and agriculture, and small rivers. PCA_5 km_3 represents sites surrounded by sloped highland areas with savannah, and large swampy rivers. PCA_5 km_4 represents sites surrounded by flat lowland areas with savannah and small rivers. PCA_5 km_5 represents sites surrounded by flat highlands with urban and agriculture, and large rivers. PCA_5 km_6 represents sites surrounded by lowland hills, with small rivers and many small basins, in unforested environment.
(DOC)

Table S3 Pearson product R correlation coefficients in the wet season model. Stepwise selection selected 3 components, none of which were correlated.
(DOC)

Table S4 Pearson product R correlation coefficients in the dry season model. Stepwise selection selected 6 components, none of which were correlated.
(DOC)

Table S5 Contingency table describing model performance of niche models constructed in Cameroon and predicted into French Guiana. The rows ‘Prediction’ are model predictions, ‘Test’ are the results from qPCR of the sites in French Guiana. Values in blue are true positives and true negatives; values in red are false positives and false negatives.
(DOC)
