# Supplementary material for: Topography and Land Cover of Watersheds Predicts the Distribution of the Environmental Pathogen Mycobacterium ulcerans in Aquatic Insects
Source: PLoS Negl Trop Dis. 2014 Nov 6;8(11):e3298. doi: 10.1371/journal.pntd.0003298 (PMC4222759; doi:10.1371/journal.pntd.0003298)
Supplement: Figure S1 — GLMulti output, for binomial and Gaussian models. Sum of absolute model residuals are plotted against AICc. Within the region of 2 AICc scores of the best model (vertical lines) we select the model with the lowest residuals (highlighted in red). (DOC) [file pntd.0003298.s001.doc]

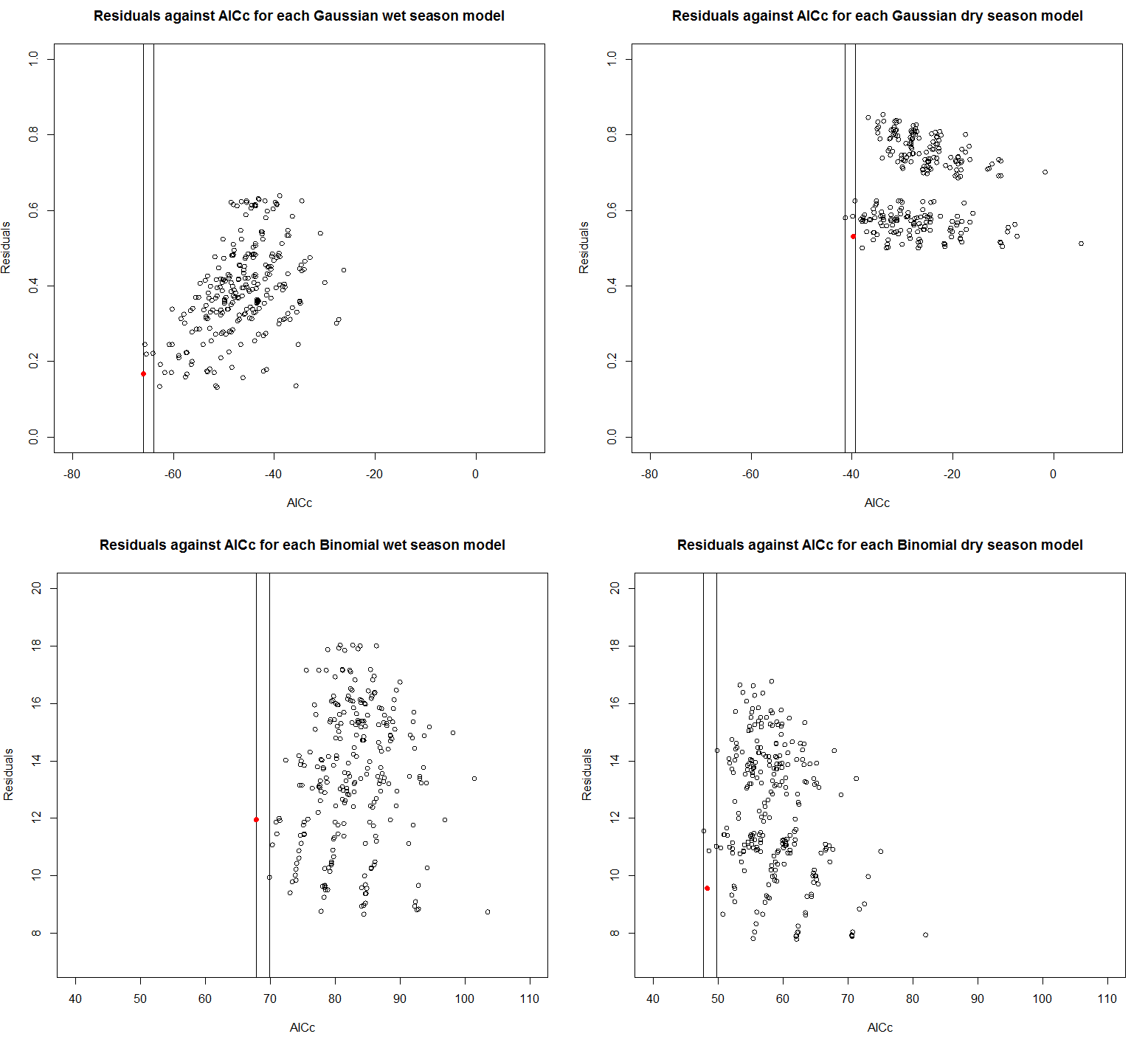


Supplementary Figure 1. GLMulti output, for binomial and Gaussian models. Sum of absolute model residuals are plotted against AICc. Within the region of 2 AICc scores of the best model (vertical lines) we select the model with the lowest residuals (highlighted in red).
