# Supplementary material for: Topography and Land Cover of Watersheds Predicts the Distribution of the Environmental Pathogen Mycobacterium ulcerans in Aquatic Insects
Source: PLoS Negl Trop Dis. 2014 Nov 6;8(11):e3298. doi: 10.1371/journal.pntd.0003298 (PMC4222759; doi:10.1371/journal.pntd.0003298)
Supplement: Figure S3 — Quantile-quantile plots of normality. The Gaussian and Binomial are both similarly normally distributed, though the Binomial displays a larger variance of residuals. (DOC) [file pntd.0003298.s003.doc]

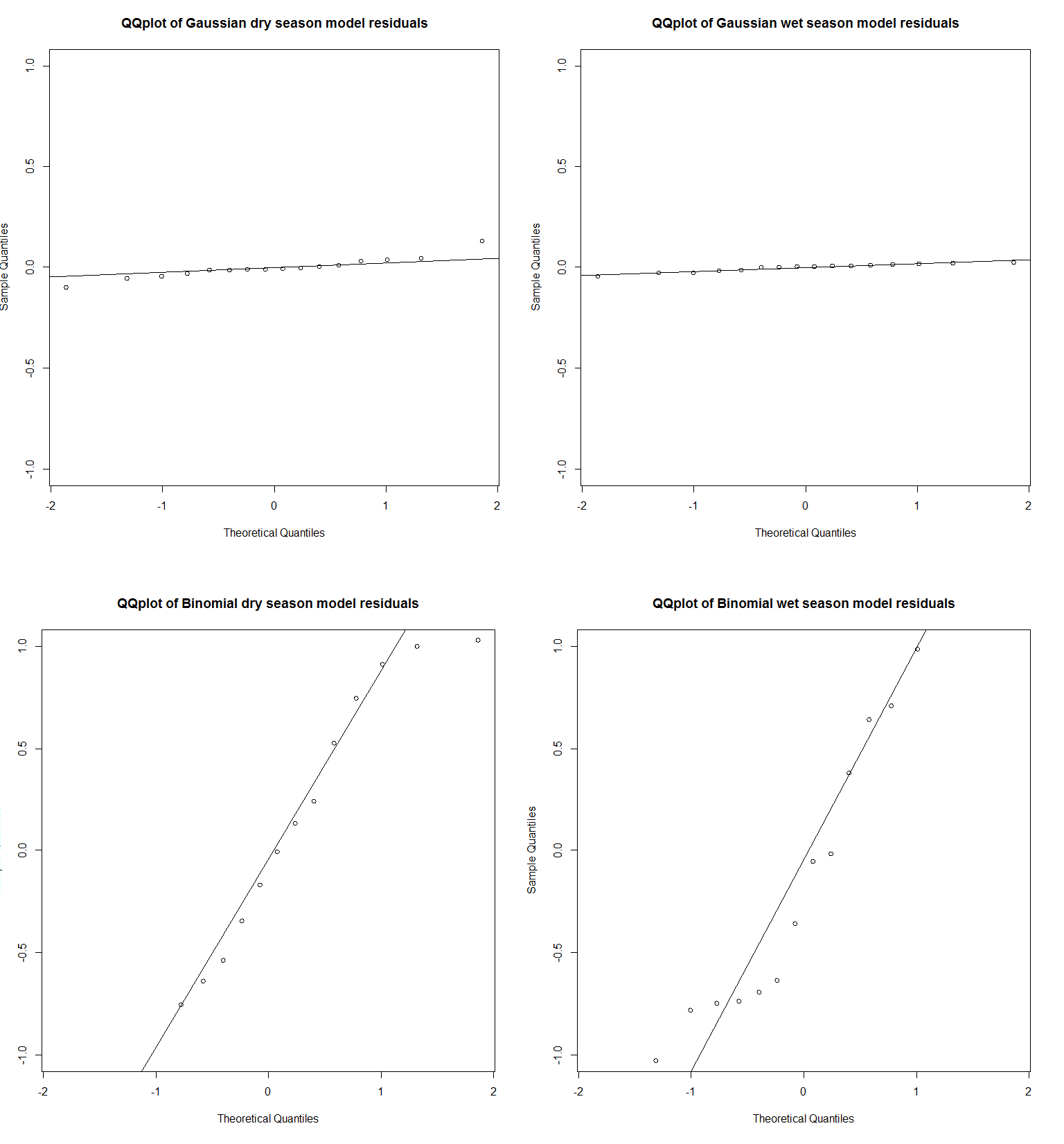


Supplementary Figure 3. Quantile-Quantile plots of normality. The Gaussian and Binomial are both similarly normally distributed, though the Binomial displays a larger variance of residuals.
