# Supplementary material for: Topography and Land Cover of Watersheds Predicts the Distribution of the Environmental Pathogen Mycobacterium ulcerans in Aquatic Insects
Source: PLoS Negl Trop Dis. 2014 Nov 6;8(11):e3298. doi: 10.1371/journal.pntd.0003298 (PMC4222759; doi:10.1371/journal.pntd.0003298)

Supplementary Table 1. Results of Principle Component Analysis for topographical and landcover variables in a watershed buffer. 95% of the variance in the data was described with 9 components, the eigenvalue of each component is given at the bottom of the table. Each component correlates differently to different variables, red highlights negative correlations, blue highlights positive correlations. PCA_ws_1 describes large watersheds that drain flood plains and swamps, with few urban and agricultural areas. These are high elevation areas with variable slopes. PCAws2 describes large watersheds that drain agriculture at flat highland areas. PCA_ws_3 describes large rivers that drain urban and agriculture areas at flat lowlands with, with little forest. PCA_ws_4 describes small rivers, with small watersheds that drain forest and swamp areas, without urban areas. These are at intermediate elevations, with flat areas. PCA_ws_5 describes small rivers that drain urban and savannah areas, predominantly in higher elevation flat lands. PCA_ws_6 corresponds to small low order streams that drain urban and forest (not agriculture) in high elevation slopes. PCA_ws_7 is larger watersheds that drain forest, savannah flood plain and swamp, in areas with flat, wet, lowlands. PCA_ws_8 represents small watersheds that drain urban & agriculture, flood plain and savannah. These areas are wet lowlands with lots of small hills. PCA_ws_9 represents small watersheds that drain wet swamps in areas that reach from low to high elevations.


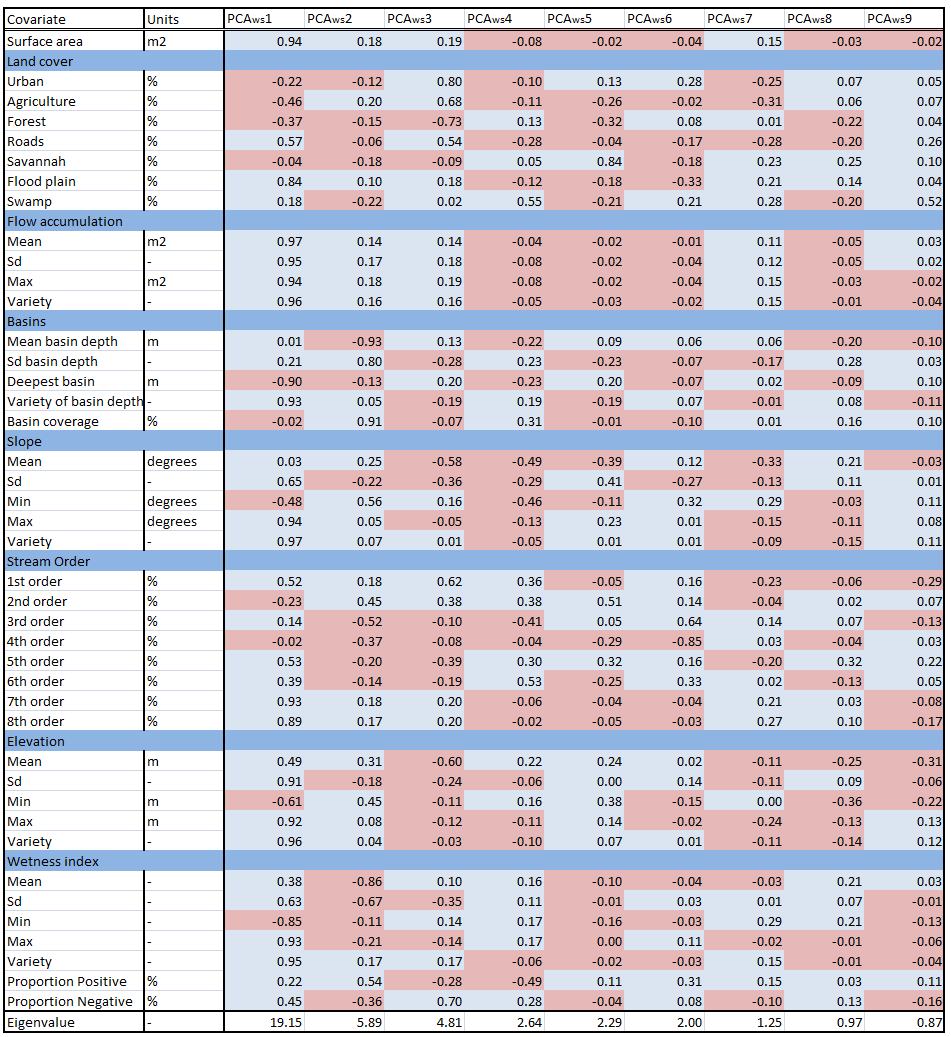

Supplement: Table S1 — Results of principle component analysis for topographical and land cover variables in a watershed buffer. 95% of the variance in the data was described with 9 components, the eigenvalue of each component is given at the bottom of the table. Each component correlates differently to different variables, red highlights negative correlations, blue highlights positive correlations. PCAws1 describes large watersheds that drain flood plains and swamps, with few urban and agricultural areas. These are high elevation areas with variable slopes. PCAws2 describes large watersheds that drain agriculture at flat highland areas. PCAws3 describes large rivers that drain urban and agriculture areas at flat lowlands with, with little forest. PCAws4 describes small rivers, with small watersheds that drain forest and swamp areas, without urban areas. These are at intermediate elevations, with flat areas. PCAws5 describes small rivers that drain urban and savannah areas, predominantly in higher elevation flat lands. PCAws6 corresponds to small low order streams that drain urban and forest (not agriculture) in high elevation slopes. PCAws7 is larger watersheds that drain forest, savannah flood plain and swamp, in areas with flat, wet, lowlands. PCAws8 represents small watersheds that drain urban & agriculture, flood plain and savannah. These areas are wet lowlands with lots of small hills. PCAws9 represents small watersheds that drain wet swamps in areas that reach from low to high elevations. (DOC) [file pntd.0003298.s004.doc]
