# Supplementary material for: Topography and Land Cover of Watersheds Predicts the Distribution of the Environmental Pathogen Mycobacterium ulcerans in Aquatic Insects
Source: PLoS Negl Trop Dis. 2014 Nov 6;8(11):e3298. doi: 10.1371/journal.pntd.0003298 (PMC4222759; doi:10.1371/journal.pntd.0003298)

Supplementary Table 2. Results of Principle Component Analysis for topographical and landcover variables in a 5km buffer around the sample site. 95% of the variance in the data was described with 6 components. Each component correlates differently to different variables, red highlights negative highlights, blue indicates positive correlations. Surface area is constant, at π5^2^=79km^2^. PCA_5km_1 represents sites surrounded by flat lowland areas and urban, agriculture and the flood plains of large rivers. PCA_5km_2 represents sites surrounded by sloped highland areas and urban and agriculture, and small rivers. PCA_5km_3 represents sites surrounded by sloped highland areas with savannah, and large swampy rivers. PCA_5km_4 represents sites surrounded by flat lowland areas with savannah and small rivers. PCA_5km_5 represents sites surrounded by flat highlands with urban and agriculture, and large rivers. PCA_5km_6 represents sites surrounded by lowland hills, with small rivers and many small basins, in unforested environment.


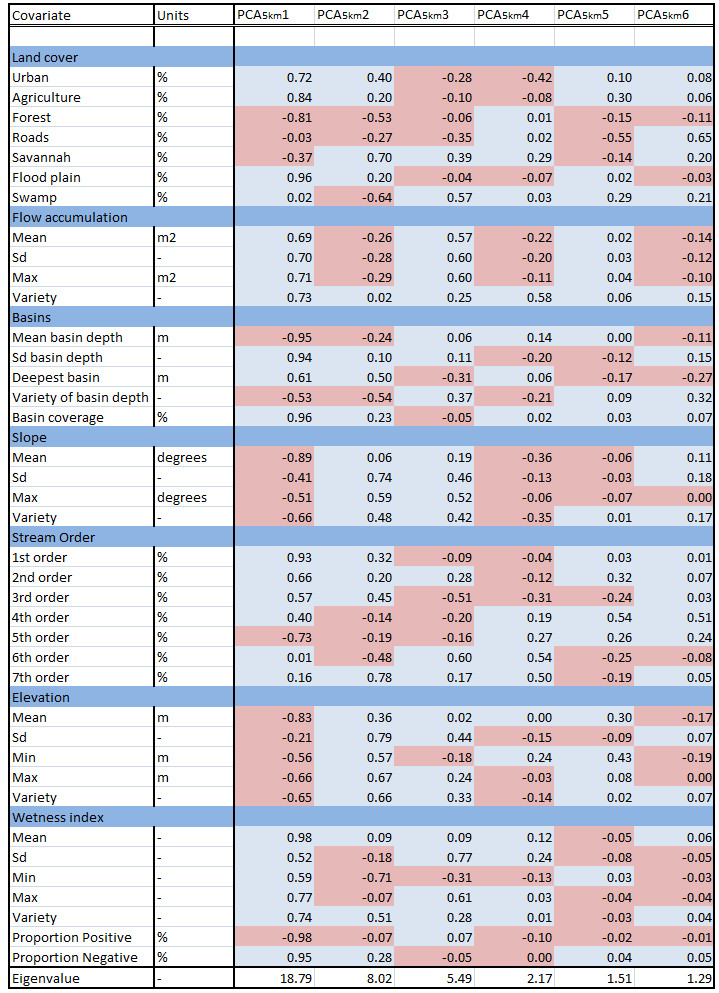

Supplement: Table S2 — Results of principle component analysis for topographical and land cover variables in a 5 km buffer around the sample site. 95% of the variance in the data was described with 6 components. Each component correlates differently to different variables, red highlights negative highlights, blue indicates positive correlations. Surface area is constant, at π52 = 79 km2. PCA5 km1 represents sites surrounded by flat lowland areas and urban, agriculture and the flood plains of large rivers. PCA5 km2 represents sites surrounded by sloped highland areas and urban and agriculture, and small rivers. PCA5 km3 represents sites surrounded by sloped highland areas with savannah, and large swampy rivers. PCA5 km4 represents sites surrounded by flat lowland areas with savannah and small rivers. PCA5 km5 represents sites surrounded by flat highlands with urban and agriculture, and large rivers. PCA5 km6 represents sites surrounded by lowland hills, with small rivers and many small basins, in unforested environment. (DOC) [file pntd.0003298.s005.doc]
