# Supplementary material for: Topography and Land Cover of Watersheds Predicts the Distribution of the Environmental Pathogen Mycobacterium ulcerans in Aquatic Insects
Source: PLoS Negl Trop Dis. 2014 Nov 6;8(11):e3298. doi: 10.1371/journal.pntd.0003298 (PMC4222759; doi:10.1371/journal.pntd.0003298)
Supplement: Table S3 — Pearson product R correlation coefficients in the wet season model. Stepwise selection selected 3 components, none of which were correlated. (DOC) [file pntd.0003298.s006.doc]

Supplementary Table 3. Pearson product R correlation coefficients in the wet season model. Stepwise selection selected 3 components, none of which were correlated.

|  | PCAws4 | PCAws9 | PCA5km2 |
| --- | --- | --- | --- |
| PCAws4 | 1 | 0 | 0.13 |
| PCAws9 | 0 | 1 | -0.16 |
| PCA5km2 | 0.13 | -0.16 | 1 |
