# Supplementary material for: Topography and Land Cover of Watersheds Predicts the Distribution of the Environmental Pathogen Mycobacterium ulcerans in Aquatic Insects
Source: PLoS Negl Trop Dis. 2014 Nov 6;8(11):e3298. doi: 10.1371/journal.pntd.0003298 (PMC4222759; doi:10.1371/journal.pntd.0003298)
Supplement: Table S4 — Pearson product R correlation coefficients in the dry season model. Stepwise selection selected 6 components, none of which were correlated. (DOC) [file pntd.0003298.s007.doc]

Supplementary Table 4. Pearson product R correlation coefficients in the dry season model. Stepwise selection selected 6 components, none of which were correlated.

|  | PCAws1 | PCAws5 | PCAws6 | PCAws8 | PCA5km2 | PCA5km4 |
| --- | --- | --- | --- | --- | --- | --- |
| PCAws1 | 1 | 0 | 0 | 0 | -0.15 | -0.03 |
| PCAws5 | 0 | 1 | 0 | 0 | 0.71 | 0.25 |
| PCAws6 | 0 | 0 | 1 | 0 | 0.11 | 0.08 |
| PCAws8 | 0 | 0 | 0 | 1 | 0.11 | -0.48 |
| PCA5km2 | -0.15 | 0.71 | 0.11 | 0.11 | 1 |  |
| PCA5km4 | -0.03 | 0.25 | 0.08 | -0.48 | 0 | 1 |
