# Supplementary material for: Topography and Land Cover of Watersheds Predicts the Distribution of the Environmental Pathogen Mycobacterium ulcerans in Aquatic Insects
Source: PLoS Negl Trop Dis. 2014 Nov 6;8(11):e3298. doi: 10.1371/journal.pntd.0003298 (PMC4222759; doi:10.1371/journal.pntd.0003298)
Supplement: Table S5 — Contingency table describing model performance of niche models constructed in Cameroon and predicted into French Guiana. The rows ‘Prediction’ are model predictions, ‘Test’ are the results from qPCR of the sites in French Guiana. Values in blue are true positives and true negatives; values in red are false positives and false negatives. (DOC) [file pntd.0003298.s008.doc]

Supplementary Table 5. Contingency table describing model performance of niche models constructed in Cameroon and predicted into French Guiana. The rows ‘Prediction’ are model predictions, ‘Test’ are the results from qPCR of the sites in French Guiana. Values in blue are true positives and true negatives; values in red are false positives and false negatives.

|  |  | Test |  |  |  |
| --- | --- | --- | --- | --- | --- |
|  |  | positive | negative |  |  |
| Prediction | positive | 3 | 2 | Sensitivity | 0.60 |
|  | negative | 9 | 4 | Specificity | 0.31 |
|  |  | PPV | NPV |  |  |
|  |  | 0.25 | 0.66 | Accuracy | 0.39 |
